# Old and new strategies for the prevention of nosocomial infections

**DOI:** 10.1186/1824-7288-41-S1-A43

**Published:** 2015-09-24

**Authors:** Ilaria Stolfi, Carla Fassi, Roberto Pedicino, Luigi Giannini

**Affiliations:** 1 Department of Obstetrics and Gynecology, Newborn Emergency Transport Service (STEN), Umberto I Policlinico of Rome, University hospital, Sapienza University of Rome, 00161, Italy; 2 Department of Obstetrics and Gynecology, Neonatal Intensive Care Unit, Umberto I Policlinico of Rome, University hospital, Sapienza University of Rome, 00161, Italy; 3 Department of Pediatrics, Umberto I Policlinico of Rome, University hospital, Sapienza University of Rome, 00161, Italy

## 

Nosocomial infections are a significant issue of public health. In Italy, the incidence of nosocomial infections range between 5 and 8% [1]; in Neonatal Intensive Care Unit (NICU) range between 7 and 24.5% [2].

Nosocomial infection in a newborn is defined as an infection arised after 48-72 hours of hospitalization. The extremely low birth weight (ELBW) neonates have an increased risk of developing infections (40%)[2], due to the immaturity of the immune system, the prolonged length of hospitalization and the frequent need for invasive procedures (central venous catheters - CVC, mechanical ventilation, parenteral nutrition, prolonged antibiotic therapies). In NICU, sepsis accounted for 45-55% of cases of nosocomial infections, followed by the lower respiratory tract infections (16-33%), skin and soft tissue infections (26.3%), urinary tract infections (8-19%) and meningitis (9.6%) [2]. The gram-positive bacteria are responsible for 65% of infections (Coagulase-negative Staphylococci - CoNS, Staphylococcus aureus and Enterococcus spp respectively in 50, 35 and 6% of cases), followed by Gram-negative bacteria (Klebsiella, Pseudomonas, E. Coli ) and fungi in 25% of cases each. Candida albicans is involved in 50% of cases of fungal infections. Viruses are accountable for epidemics in the NICU, but the incidence of viral infections is likely to be underestimated.

The prevention of nosocomial infections is an essential element for the management of the newborns [3,4] and is based on strategies to reduce the risk factors related to the newborn (immune system, carefull skin care, etc.) and to improve the invasive care procedures (implementation and dissemination of guide lines for accurate and proper hand hygiene [4,5], for prevention of CVC related infections [4,6] and ventilator-associated pneumonia [7], promotion of enteral feeding with breast milk [8]). Not least, the need for accurate diagnostic strategies for early detection of neonatal infections and a rational use of antimicrobial therapies and antibiotic prophylaxis [9,10]. The new strategies of prophylaxis of infections involving the use of bioactive substances with anti-infective properties, such as lactoferrin [11]; the use of probiotics, which have recognized immunomodulatory and anti-infectious activities [12]; the prophylaxis with antifungal drugs [13]. Lastly, NICU should also meet specific criteria of organization, providing to maintain an adequate ratio nurses/beds, avoid overcrowding and understaffing, make easily available devices for hand washing, organize meetings for training/provide to caregivers regular feedback of performance data, plan continuous monitoring and a surveillance system of the rate of nosocomial infections and avoid preventive measures of unproven effectiveness. 

## References

[B1] Istituto Superiore di Sanità Infezioni correlate all'assistenza. Aspetti epidemiologici http://www.epicentro.iss.it/problemi/infezioni_correlate/epid.asp.2009

[B2] Stronati M Sepsi Neonatali: nuove strategie preventive Minerva Pediatr 2013 65 103 110 23422580

[B3] Garland JS Uhing MR Strategies to prevent bacterial and fungal infection in the neonatal intensive care unit Clin Perinatol 2009 36 1 1 13 10.1016/j.clp.2008.09.005 19161861

[B4] Polin RA Denson S Brady MT Committee on Fetus and Newborn; Committee on Infectious Diseases. Strategies for prevention of health care-associated infections in the NICU Pediatrics 2012 129 e 1085 1093 10.1542/peds.2012-0145 22451712

[B5] World Health Organization Guidelines on hand hygiene in health care: First global patient safety challenge clean care is safer care” 2009 WHO 23805438

[B6] CDC Guidelines for the Prevention of Intravascular Catheter-Related Infections MMWR 2002 51

[B7] CDC Guidelines for the Preventing Health-Care-Associated Pneumonia 2003

[B8] Adamkin DH Mother's milk, feeding strategies, and lactoferrin to prevent necrotizing enterocolitis JPEN J Parenter Enteral Nutr 2012 36 1, Suppl 25S 29S 22237873 10.1177/0148607111420158

[B9] Isaacs D Unnatural selection: reducing antibiotic resistance in neonatal units Arch Dis Child Fetal Neonatal Ed 2006 91 F 72 74 10.1136/adc.2005.074963 PMC2672659 16371394

[B10] Fanos V Cuzzolin L Atzei A Testa M Antibiotics and antifungals in neonatal intensive care units: a review J Chemother 2007 19 5 20 10.1179/joc.2007.19.1.5 17309846

[B11] Manzoni P Italian Task Force for the Study and Prevention of Neonatal Fungal Infections-the Italian Society of Neonatology. Bovine lactoferrin prevents invasive fungal infections in very low birth weight infants: a randomized controlled trial Pediatrics 2012 129 116 123 10.1542/peds.2011-0279 22184648

[B12] Alfaleh K Anabrees J Bassler D Al-Kharfi T Probiotics for prevention of necrotizing enterocolitis in preterm infants Cochrane Database Syst Rev 2011 3 CD005496 21412889 10.1002/14651858.CD005496.pub3

[B13] Manzoni P De Luca D Stronati M Jacqz-Aigrain E Ruffinazzi G Luparia M Tavella E Boano E Castagnola E Mostert M Farina D Prevention of nosocomial infections in neonatal intensive care units Am J Perinatol 2013 30 2 81 88 10.1055/s-0032-1333131 23292914

